# Synthesis of Cubic Ni(OH)_2_ Nanocages Through Coordinating Etching and Precipitating Route for High-Performance Supercapacitors

**DOI:** 10.1186/s11671-019-3096-6

**Published:** 2019-08-02

**Authors:** Liangliang Tian, Tong Yang, Wanrong Pu, Jinkun Zhang

**Affiliations:** 1 Research Institute for New Materials Technology, Chongqing University of Arts and Sciences, Chongqing, People’s Republic of China; 2 Faculty of Materials and Energy, Southwest University, Chongqing, People’s Republic of China; 3 School of Pharmacy, Tianjin Medical University, Tianjin, People’s Republic of China; 4 College of Pharmaceutical Science, Zhejiang University of Technology, Zhejiang, People’s Republic of China

**Keywords:** Transition metal hydroxides, Ni(OH)_2_, Nanocages, Coordinating etching and precipitating, Supercapacitor, Energy storage

## Abstract

Rational design of cage-like structure is an effective method for the improvement of the capacitive performance of transition metal hydroxides. In this work, cubic Ni(OH)2 nanocages (Ni(OH)2 NCs) were constructed through a coordinating etching and precipitating (CEP) route. Ni(OH)2 NCs possess abundant active sites, sufficient diffusion channels, and accelerated electron transfer rate, which are beneficial for electrochemical kinetics. As a positive electrode for supercapacitors, the Ni(OH)2 NCs/Ni foam (NF) electrode presents a high specific capacitance of 539.8 F g^−1^ at 1 A g^−1^, which is much larger than that of broken Ni(OH)2 NCs/NF (Ni(OH)2 BNCs/NF, 87.3 F g^−1^ at 1 A g^−1^). In addition, the Ni(OH)2 NCs/NF electrode still retains 96.9% of its initial specific capacitance after 2000 cycles. The asymmetric supercapacitor (ASC) devices were assembled using Ni(OH)2 NCs/NF and activated carbon (AC)/NF as positive and negative electrodes, respectively. The ASC exhibits a higher energy density of 23.3 Wh kg^−1^ at a power density of 800 W kg^−1^ compared to Ni(OH)2 BNCs/NF (3 Wh kg^−1^ at 880 W kg^−1^). These results demonstrate that the Ni(OH)2 NCs/NF electrode presents potential applications in the field of energy storage. The design of cage-like structure paves an effective way to achieve high-performance electrode materials.

**Electronic supplementary material:**

The online version of this article (10.1186/s11671-019-3096-6) contains supplementary material, which is available to authorized users.

## Background

To overcome the challenges of environment pollution and energy crisis, there are significant demands to develop safe, renewable, clean, and high-performance energy storage devices as alternatives to fossil fuels [1, 2]. Supercapacitors possess excellent characteristics to meet these issues, such as high-power capability (10–20 times that of batteries), high-rate performance, short charging time, and environment-friendly nature [3, 4]. Electric double-layer capacitors (EDLCs) and pseudocapacitors (PCs) are the commonly researched two types of supercapacitors. Thereinto, PCs governed by redox on/near the surface of transition metal oxide/hydroxide electrodes always have higher energy density than EDLCs and have become the hot issues in this field [5–10]. As a typical transition metal hydroxide, Ni(OH)_2_ was reported as a high-performance electrode material for PCs due to the redox couple of Ni^3+^/Ni^2+^ in an alkaline medium [11, 12]. Nevertheless, the acquired specific capacitance of Ni(OH)_2_ is always much lower than the theoretical value because of the insufficient utilization of electrode materials.

Inspired by kinetics, the capacitive performance of electrode materials can be mediated through the design of microstructure and morphology. Tremendous efforts have been devoted to the synthesis of Ni(OH)_2_ electrode materials with unique microstructures to achieve high-efficiency storage performance [13, 14]. Thereinto, constructing cage-like hollow porous structure was regarded as an effective method to obtain high-performance electrodes. Specifically, a cage-like structure can make full use of the inner and outer surface area and provide enough redox-active sites, leading to enhanced specific capacitance. Furthermore, the porous shell affords amounts of diffusion paths for electrolyte, which is beneficial to the reversibility of the electrode, resulting in excellent cycling stability and high-rate performance. Regarding electron transfer kinetics, the nanosized thin shell refines the transfer route of electrons and accelerates the electron transfer rate [15, 16]. Thus, higher capacitive performance of Ni(OH)_2_ can be obtained through the design of cage-like hollow porous architecture.

The templated chemical process is the commonly used method to prepare cage-like architectures [17, 18]. The final products can accurately duplicate the geometrical shape of the templates and retain a well-defined morphology with narrow size distribution [19, 20]. In this work, Ni(OH)_2_ NCs were fabricated using cubic Cu_2_O crystals as sacrificial templates through thiosulfate involved coordinating etching and precipitating (CEP) principle. The synthesized Ni(OH)_2_ NCs/NF was employed as positive electrode for supercapacitors and Ni(OH)_2_ BNCs/NF was introduced as a contrast sample to confirm the structural advantages of cage-like architecture. Ni(OH)_2_ NCs/NF displays a high specific capacitance of 539.8 F g^−1^ at 1 A g^−1^, which is much larger than that of Ni(OH)_2_ BNCs/NF (87.3 F g^−1^ at 1 A g^−1^). The asymmetric supercapacitor (ASC) device presents a high energy density of 23.3 Wh kg^−1^ at 800 W kg^−1^, and this value is much larger than that of Ni(OH)_2_ BNCs/NF//AC (3 Wh kg^−1^ at 880 W kg^−1^). The results reveal that Ni(OH)_2_ NCs/NF electrode exhibits an attractive prospect in supercapacitors. The way to design cage-like hollow porous architecture is also meaningful in other fields, such as sensors and catalysts.

## Methods/Experimental

### Preparation of Cu_2_O Templates

Cubic Cu_2_O crystals were synthesized according to our previous report [21]. Fifty milliliters NaOH solution (2 M) was added into the stirred CuCl_2_·2H_2_O (500 ml, 0.01 M) within 3 min at 55 °C. After stirring for 30 min, 50 mL 0.6 M ascorbic acid solution was added dropwise. The final samples were centrifugated after 3 h and dried in a vacuum.

### Synthesis of Ni(OH)_2_ NCs

400 mg Cu_2_O templates and different dosage of NiCl_2_ powers were poured into a 1000-mL beaker containing 400 mL mixed water and alcohol (volume ratio = 1:1). The mass ratio of Cu_2_O templates and NiCl_2_ powers is controlled as 5:1, 2.5:1, 1.67:1, and 1.25:1 (corresponding NiCl_2_ dosage is 80 mg, 160 mg, 240 mg, and 320 mg, respectively). After ultrasonic treatment for 10 min, 13.2 mg polyvinyl pyrrolidone (PVP) was dispersed into the solution under stirring. After 30 min, 160 mL 1 M Na_2_S_2_O_3_ was added dropwise into the solution at room temperature. After 3 h, the final products were collected by centrifugation and dried in an oven. Ni(OH)_2_ BNCs were obtained through the ultrasonic treatment of Ni(OH)_2_ NCs for 2 h in alcohol (Additional file 1: Figure S1).

### Materials Characterizations

The structure and chemical composition of the products were analyzed by X-ray powder diffraction (XRD, Rigaku D/Max-2400) using Cu Kα radiation and ESCALAB 250Xi X-ray photoelectron spectroscopy (XPS, USA). The morphologies of the products were investigated on a Zeiss Gemini 300 field emission scanning electron microscopy (FESEM). Transmission electron microscope (TEM) observations were conducted on a FEI F20 device. The specific surface area and porous feature were measured on a Belsort-max instrument.

### Electrochemical Measurements

All the electrochemical measurements were performed on a μIII Autolab workstation in 3 M KOH with Pt foil (1 cm × 1 cm) and Ag/AgCl (saturated KCl) as counter and reference electrodes, respectively. The working electrodes were constructed by the following procedures: first, the electrode materials (Ni(OH)_2_ NCs obtained at different reaction time and Ni(OH)_2_ BNCs), acetylene black, and polytetrafluoro ethylene (5% PTFE) were mixed together with a mass ratio of 80:15:5 in ethanol. And then, the mixture was coated onto NF (1 cm × 1 cm) and dried in an oven. The loading mass was calculated as 3.4 mg/cm^2^. The electrochemical performance was examined by cyclic voltammetry (CV), galvanostatic charge-discharge (GCD), and electrochemical impedance (EIS). The EIS tests were performed between 0.01 and 100 kHz with a perturbation amplitude of 5 mV. The specific capacitance of the electrodes was calculated according to the following equation:

1 $$ C=\frac{I\varDelta t}{m\varDelta V} $$

where *I* is the discharge current (A), *t* is the discharge time (s), Δ*V* is the potential window (V), *m* is the total mass (g) of electrode materials. The ASCs were prepared with Ni(OH)_2_ NCs (or Ni(OH)_2_ BNCs)) and AC as the positive and negative electrodes, respectively. The AC electrode was prepared by coating a mixture of AC and PTFE binder (90:10) on NF (1 cm × 1 cm). Then the two electrodes were assembled together with a separator in 3 M KOH.

## Results and Discussions

### Characterizations

**Fig. 1:**
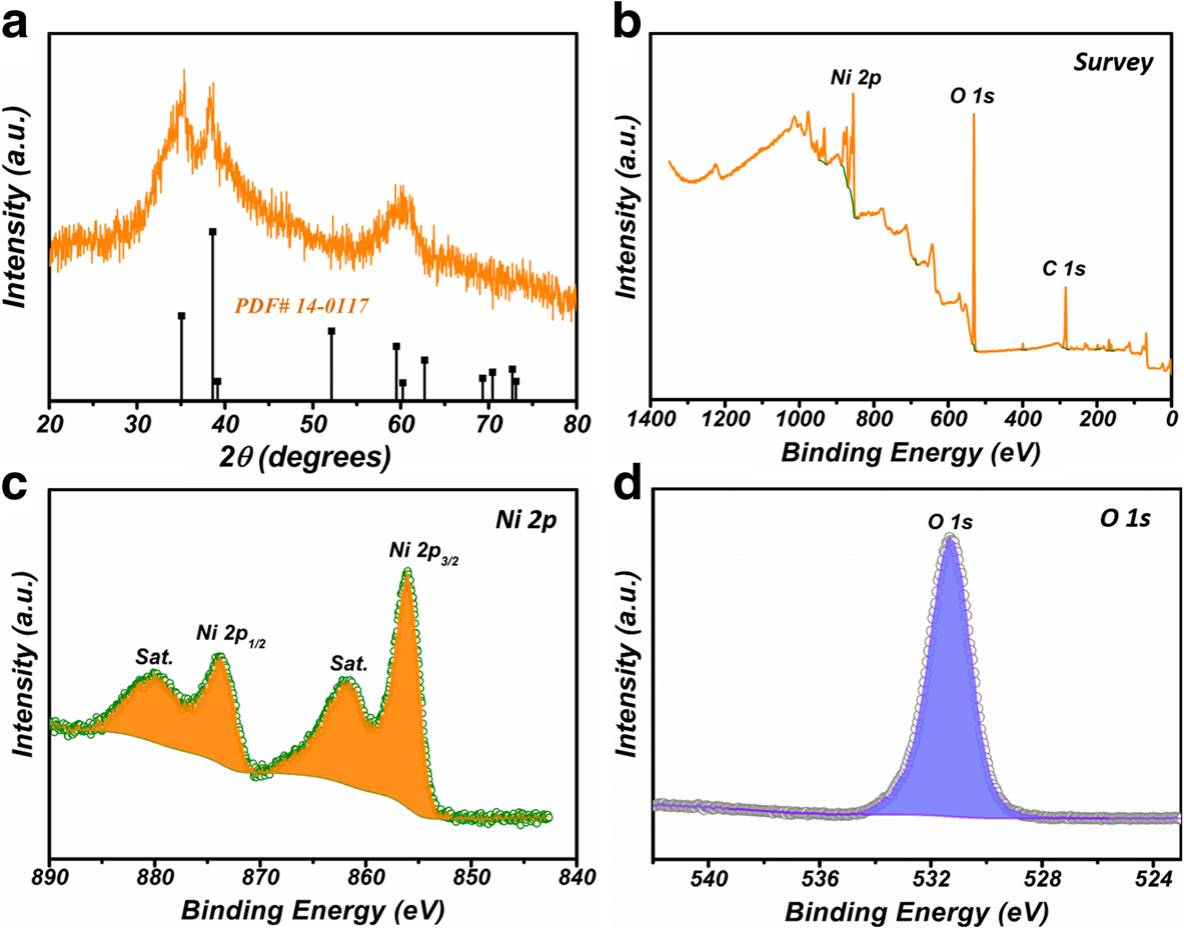
**a** XRD pattern of the prepared Ni(OH)_2_ NCs. **b**–**d** XPS spectra of the Ni(OH)_2_ NCs. **b** Survey. **c** Ni 2p. **d** O 1s

XRD pattern of the prepared Ni(OH)_2_ NCs was recorded in Fig. 1a. The observed three strong peaks located at 33.1°, 38.5°, and 60.2° correspond to (100), (101), and (003) crystalline planes of hexagonal *β*-Ni(OH)_2_ (JCPDS no. 14-0117) [22]. XPS measurements were conducted to confirm the chemical composition. Ni, O, and C signals are clearly observed in the survey spectrum, revealing that the sample is mainly composed of Ni and O. As displayed in Fig. 1c, the concentrated signals located at 873.7 eV and 856.1 eV with a separation of 17.6 eV can be attributed to Ni 2p_1/2_ and Ni 2p_3/2_ of Ni^2+^_,_ respectively [23, 24]. The peaks located at 879.9 eV and 861.7 eV are the corresponding satellite signals for Ni 2p_1/2_ and Ni 2p_3/2,_ respectively. As shown in Fig. 1d, the O1s peak located at 531.2 eV presents a typical feature of Ni-O-Ni bond in Ni(OH)_2_ [25, 26]. On the basis of the above discussions, the as prepared products can be deduced to Ni(OH)_2_ phase.

**Fig. 2:**
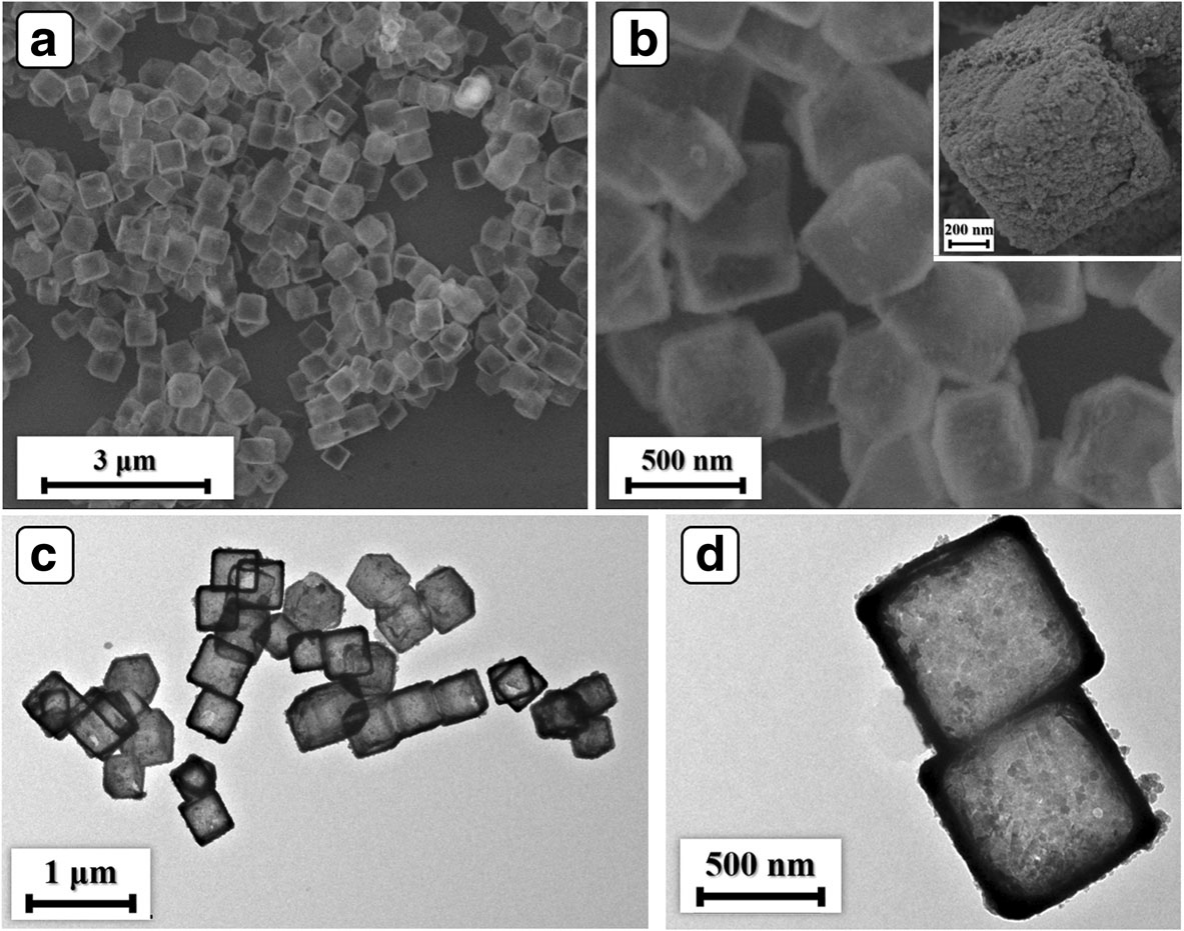
**a**, **b** SEM and **c**, **d** TEM images of the Ni(OH)_2_ NCs

SEM and TEM observations were employed to further confirm the morphology feature of the products. Additional file 1: Figure S2a displays the XRD pattern of the prepared Cu_2_O. All the diffraction peaks can be indexed to JCPDS no. 78-2076, confirming the successful preparation of Cu_2_O. SEM image of Cu_2_O templates in Additional file 1: Figure S2b reveals cubic feature of the products with an edge length about 500 nm. As noticed in Fig. 2a, the Ni(OH)_2_ samples retain uniform well-defined cubic morphology after the CEP process. The Ni(OH)_2_ cubes have an edge length of 500 nm (Fig. 2b), which is more or less the same as Cu_2_O templates. As can be seen from the inset of Fig. 2b, the surface of Ni(OH)_2_ cubes is composed of quantities of fine particles and presents porous characteristic. The TEM image in Fig. 2c exhibits apparent internal cavity, revealing the cage-like feature of Ni(OH)_2_ products. As displayed in Fig. 2d, the edge length is of 500 nm, which is consistent with the observation of SEM. Moreover, the shell thickness of Ni(OH)_2_ NCs is identified as 50 nm (Fig. 2d). The investigations of SEM and TEM demonstrate the cage-like feature of the products. The cage-like hollow porous structure provides a large surface area and amounts of diffusion paths, which may favor the mass transport process, leading to outstanding capacitive performance.

**Fig. 3:**
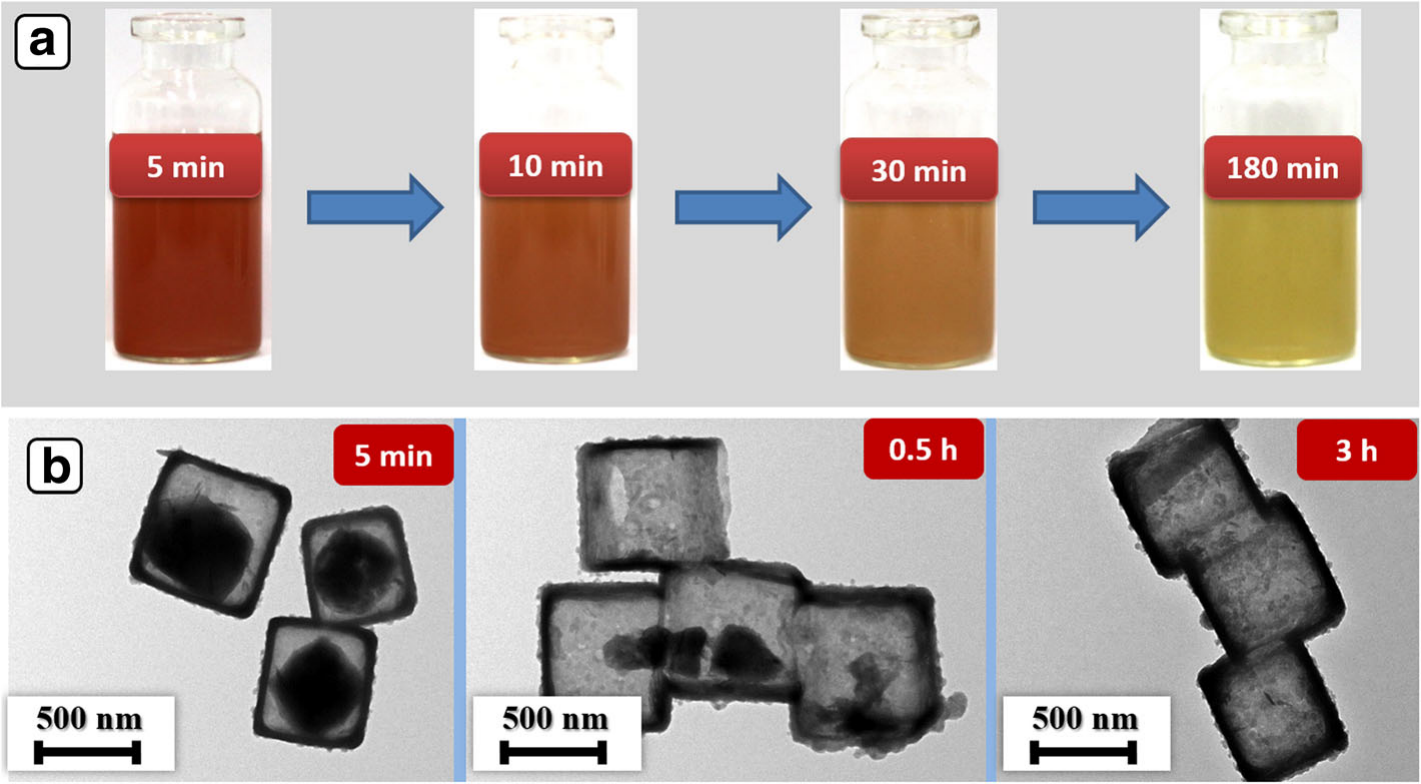
**a** The optical photographs of the reaction system at a different reaction time. **b** TEM images of the products obtained at different reaction time

**Scheme 1:**
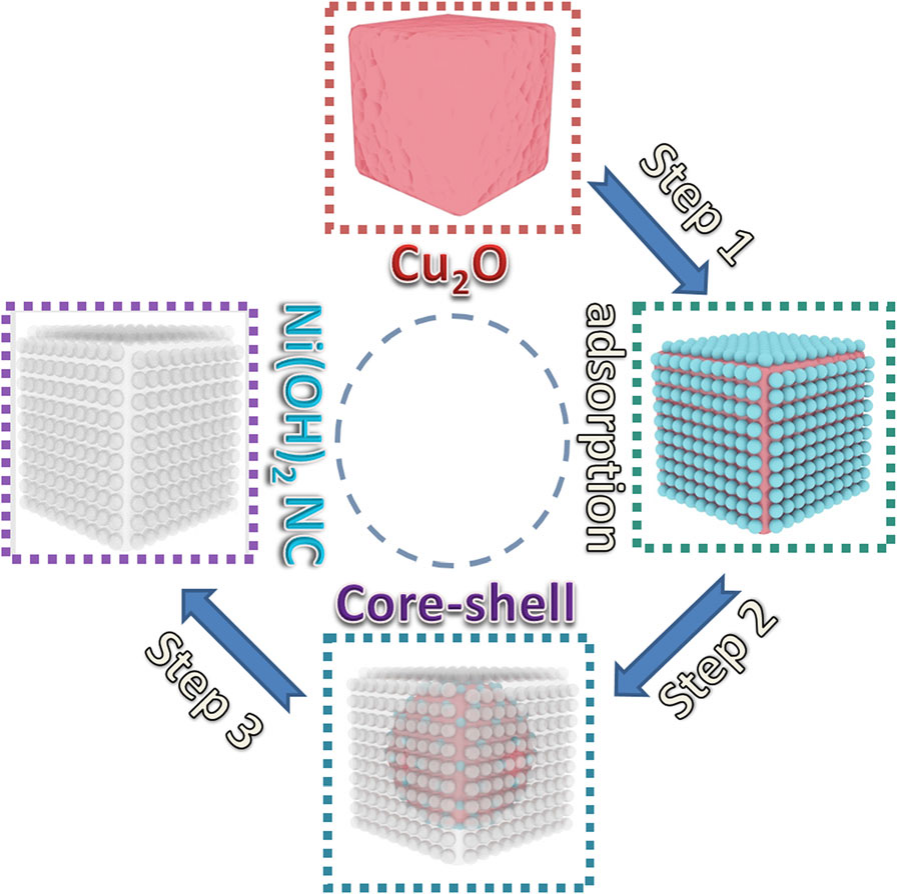
Schematic diagram for the formation process

The optical photographs and TEM images of Ni(OH)_2_ NCs were recorded at different reaction times to realize the formation mechanism. As displayed in Fig. 3a, the reaction solution exhibits brick-red color at 5 min, indicating that little reactions occur at the initial stage. Afterwards, the color of the solution gradually becomes lighter. After 3 h, the color of the solution turns into light green, which is the color of the final products. As illustrated in Fig. 3b, the products present partly hollow internal cavity due to the dissolution of Cu_2_O templates at 5 min. Moreover, the etching of internal Cu_2_O preferentially occurred at the corner owing to the adequate diffusion kinetics. The internal Cu_2_O crystals dissolve continuously until it completely disappears at 3 h. The schematic diagram was illustrated in Scheme 1. Generally, the formation mechanism of Ni(OH)_2_ NCs is shown below (Eq. (2)):

2 $$ {\mathrm{Ni}}^{2+}+2{\mathrm{OH}}^{-}\to \mathrm{Ni}{\left(\mathrm{OH}\right)}_2 $$

Ni^2+^ ions in Eq. (2) are the absorbed Ni^2+^ on the surface of Cu_2_O crystals (Step 1). OH^−^ ions in Eq. (2) are released from the corrosion of Cu_2_O crystals (Eq. (3)) and hydrolyzation of S_2_O_3_^2-^ (Eq. (4)).

3 $$ {Cu}_2O+{xS}_2{O_3}^{2-}+{H}_2O\to {\left[{Cu}_2{\left({S}_2{O}_3\right)}_x\right]}^{2-2x}+2{OH}^{-} $$

4 $$ {\mathrm{S}}_2{{\mathrm{O}}_3}^{2-}+{\mathrm{H}}_2\mathrm{O}\to {\mathrm{H}\mathrm{S}}_2{{\mathrm{O}}_3}^{2-}+{\mathrm{O}\mathrm{H}}^{-} $$

Equations (3) and (4) are the mechanism for S_2_O_3_^2−^ involved CEP process, which happens in Steps 2 and 3. The detailed kinetics process is similar to the formation of Co(OH)_2_ NCs in our published article [27]. The transport of S_2_O_3_^2−^ towards Cu_2_O determines the corrosion rate and the released OH^−^ ions from interior presents the growth rate of Ni(OH)_2_ NCs. The cooperative control of the two processes results in the formation of well-defined Ni(OH)_2_ NCs.

**Fig. 4:**
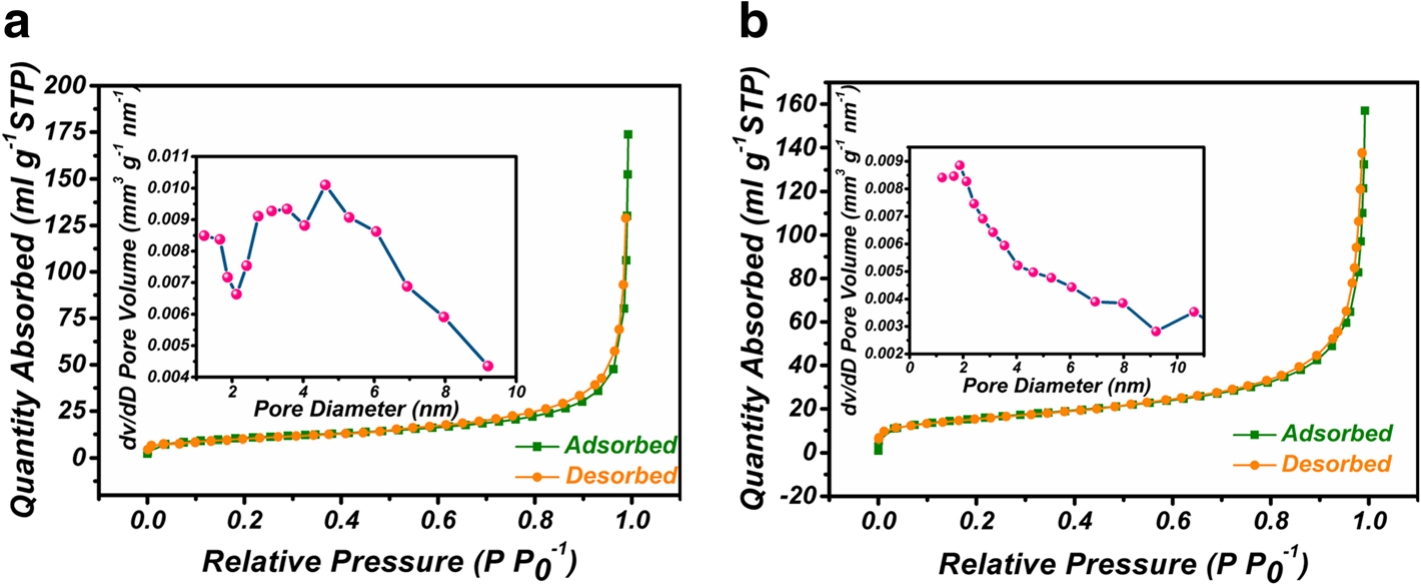
BET measurements of **a** Ni(OH)_2_ NCs and (b) Ni(OH)_2_ BNCs. Insets of **a** and **b** are the corresponding pore size distributions of Ni(OH)_2_ NCs and Ni(OH)_2_ BNCs, respectively

Fig. 4 shows the N_2_ adsorption-desorption isotherm curves of Ni(OH)_2_ NCs and Ni(OH)_2_ BNCs. The BET surface area of Ni(OH)_2_ NCs is 54.7 m^2^/g, which is much larger than that of Ni(OH)_2_ BNCs (38.1 m^2^/g). The results indicate that the hollow porous architecture endows Ni(OH)_2_ NCs with a larger specific surface area. The pore size distributions (insets of a and b) reveal the mesoporous structure of Ni(OH)_2_ NCs and Ni(OH)_2_ BNCs. The pore volume of Ni(OH)_2_ NCs is calculated as 0.25 cm^3^/g, which is larger than Ni(OH)_2_ BNCs (0.19 cm^3^/g). Furthermore, a concentrated pore distribution between 2.7 and 6.1 nm is investigated for Ni(OH)_2_ NCs, which is related to the interspace between nanoparticles. However, no obvious concentrated pore distribution is observed for Ni(OH)_2_ BNCs, revealing the destruction of ordered diffusion channels. The large surface area and ordered diffusion channels are beneficial for electrochemical kinetics, resulting in excellent capacitive performance.

### Electrochemical Performance of Ni(OH)_2_ NCs

**Fig. 5:**
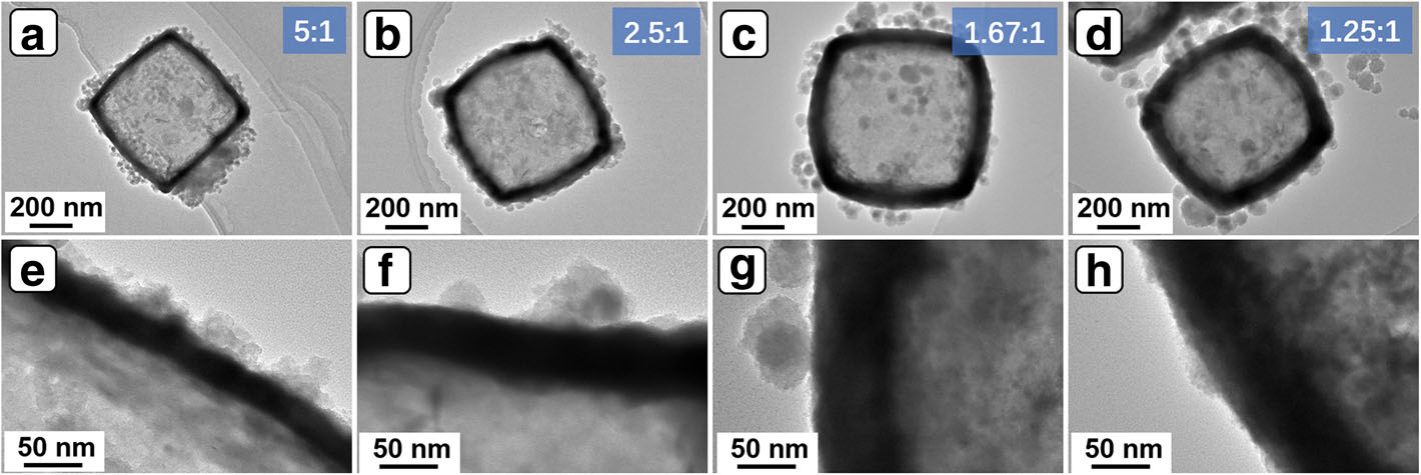
**a**–**d** TEM images of the Ni(OH)_2_ NCs obtained with different mass ratio of Cu_2_O/NiCl_2_. **e**–**h** TEM images of the corresponding shells of **a**–**d**

**Fig. 6:**
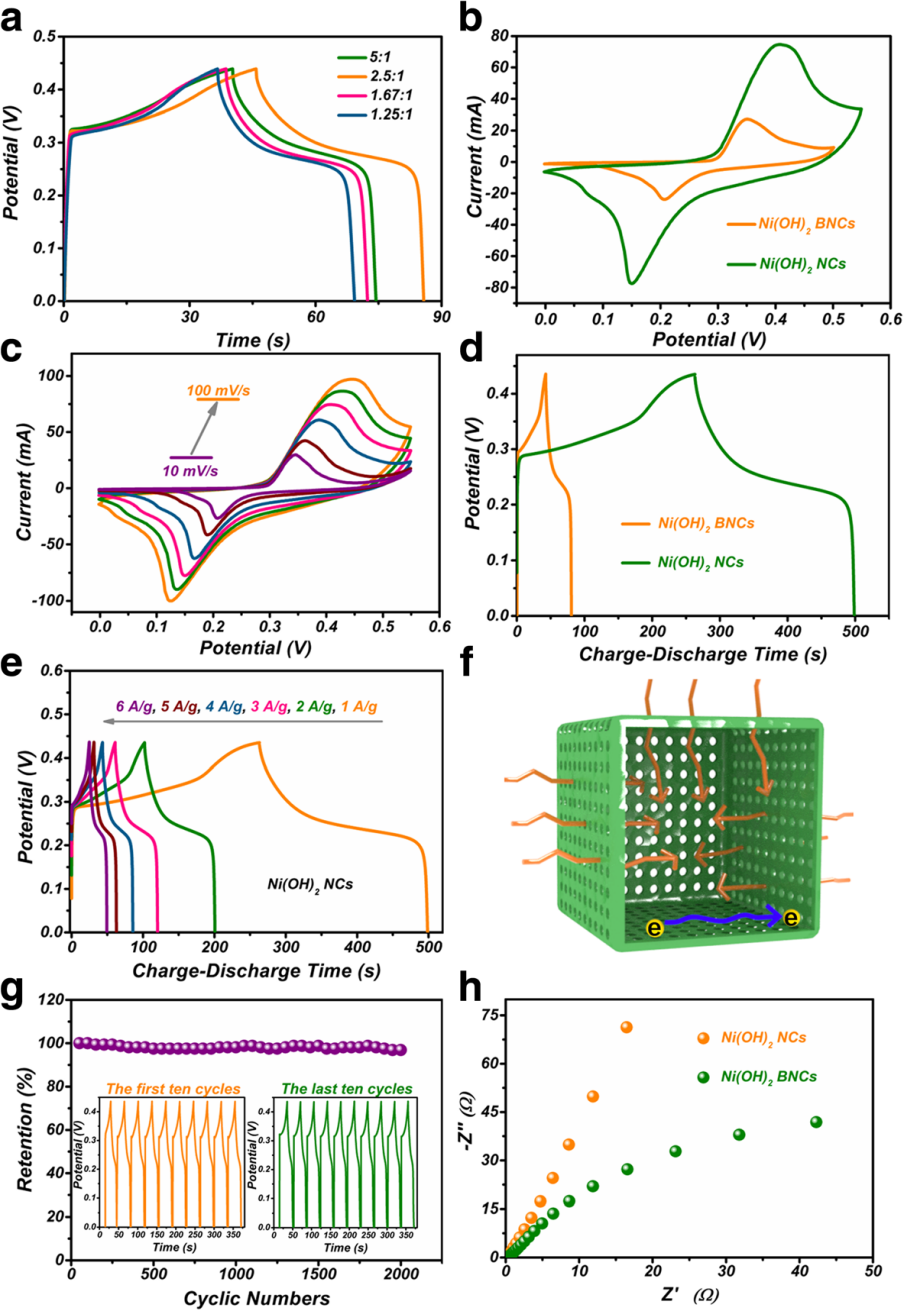
**a** GCD curves of the products obtained with different mass ration of Cu_2_O/NiCl_2_ at 4 A g^−1^. **b** The CVs of Ni(OH)_2_ NCs/NF and Ni(OH)_2_ BNCs/NF at scan rate of 60 mV/s. **c** The CVs of Ni(OH)_2_ NCs/NF at different scan rates. **d** GCD curves of Ni(OH)_2_ NCs/NF and Ni(OH)_2_ BNCs/NF at 1 A g^−1^. **e** GCD curves of Ni(OH)_2_ NCs/NF at different current densities. **f** The schematic of charge storage advantages for Ni(OH)_2_ NCs. **g** The cycling stability of Ni(OH)_2_ NCs/NF at 8 A g^−1^. **h** The EIS spectra of Ni(OH)_2_ NCs/NF and Ni(OH)_2_ BNCs/NF

In order to obtain the best capacitive property, Ni(OH)_2_ NCs with different shell thickness were prepared by controlling the dosage of NiCl_2_ powders. As shown in Fig. 5, the shell thickness apparently increases from 27.4 to 76.7 nm with the increase of mass ratio from 5:1 to 1.67:1. However, the shell thickness only slightly increases from 76.7 nm to 79 nm with the further increase of mass ratio to 1.25:1. The results can be attributed to the kinetics difficulty in mass diffusion caused by the hindering of shell. The GCD curves of Ni(OH)_2_ NCs obtained with different NiCl_2_ dosage were measured and the data were recorded in Fig. 6a. It is clear that the sample with Cu_2_O/NiCl_2_ 2.5:1 displays the longest discharge time under 4 A/g, indicating the best capacitive performance. This result can be ascribed to the suitable mass transport kinetics derived from the moderate shell thickness. Furthermore, the capacitive performance of Ni(OH)_2_ NCs obtained with Cu_2_O/NiCl_2_ 2.5:1 was contrastively evaluated with Ni(OH)_2_ BNCs. As shown in Fig. 6b, significant redox peaks are clearly observed in the CV curves of Ni(OH)_2_ NCs and Ni(OH)_2_ BNCs, revealing pseudocapacitive characteristic of the two electrodes. The redox process corresponds to the storage mechanism related to Ni(OH)_2_/NiOOH redox couple illustrated in Eq. (5) [28, 29].

5 $$ \mathrm{Ni}{\left(\mathrm{OH}\right)}_2+{\mathrm{OH}}^{-}\leftrightarrow \mathrm{Ni}\mathrm{OOH}+{\mathrm{H}}_2\mathrm{O}+{e}^{-} $$

The CV encapsulated area of Ni(OH)_2_ NCs is much larger than that of Ni(OH)_2_ BNCs, demonstrating higher specific capacitance. The CVs of Ni(OH)_2_ NCs at various scan rates are depicted in Fig. 6c. The CV curve still retains a well-defined shape even at a high scan rate of 100 mV/s, demonstrating outstanding rate capability and high electrochemical reversibility. Furthermore, the peak current linearly increases with the square root of scan rates, revealing that bulk diffusion is the dominated factor (Additional file 1: Figure S3). As presented in Fig. 6d, the GCD curves of Ni(OH)_2_ NCs show longer discharge time than Ni(OH)_2_ BNCs at 1 A g^−1^, proving that Ni(OH)_2_ NCs exhibit higher specific capacitance than Ni(OH)_2_ BNCs. Fig. 6e presents the GCD curves of Ni(OH)_2_ NCs at different current densities. The calculated specific capacitances for Ni(OH)_2_ NCs are 539.8, 445.5, 409.4, 391.3, 360.2, and 340.7 F g^−1^ at 1, 2, 3, 4, 5, and 6 A g^−1^, respectively (Additional file 1: Figure S4). Those values calculated for Ni(OH)_2_ BNCs are 87.3, 77.4, 72.9, 67.8, 64.1, and 60.5 F g^−1^ at corresponding current density (Additional file 1: Figure S5). The structural advantages for Ni(OH)_2_ NCs are illustrated in Fig. 5f. First, the cage-like feature provides quantities of active sites for Faraday reactions. Secondly, the porous thin shell shortens the migration distance of electrons, resulting in high electron transfer rate. Third, the porous shell affords sufficient diffusion channels for electrolyte, improving the utilization rate of Ni(OH)_2_. The cycling stability of Ni(OH)_2_ NCs was evaluated by repeating the GCD measurements at 8 A g^−1^ (Fig. 6 g). It is noticed that the specific capacitance still retains 96.9% of its initial value after 2000 cycles, which is much larger than that of Ni(OH)_2_ BNCs (61.5%, Additional file 1: Figure S6). As shown in the inset, the last 10 cycles show little difference compared to the first 10 charge-discharge cycles, revealing excellent stability. The little attenuation of the capacitance can be attributed to the small amount of shedding of Ni(OH)_2_ NCs from NF. The internal void and pores in the shell afford enough space for the release of a strain during the cycling process [30].

In order to confirm the advantages of cage-like structure in kinetics, EIS spectra were recorded in Fig. 6h and the equivalent circuit was illustrated in Additional file 1: Figure S7. The equivalent circuit is mainly composed of Rs, Rct, Zw, CPE, and CL. Thereinto, Rs is internal resistance of the electrode system. Rct is the charge transfer resistance related to the radius of semicircle in EIS spectra. Zw is the Warburg impendence corresponding to the slope of EIS in high frequency. Although Ni(OH)_2_ NCs/NF electrode has more or less the same Rs value (0.27 Ω) compared to Ni(OH)_2_ BNCs/NF (0.25 Ω), Ni(OH)_2_ NCs/NF has a much lower Rct (120.8 Ω) than that of Ni(OH)_2_ BNCs (976.5 Ω), revealing higher electron transfer rate. The high electron transfer rate can be attributed to enough thin shell of Ni(OH)_2_ NCs. Apparently, Ni(OH)_2_ NCs/NF electrode presents a much larger slope than Ni(OH)_2_ BNCs/NF, demonstrating more straightway diffusion process. The unimpeded diffusion can be ascribed to the ordered channels and porous characteristic of Ni(OH)_2_ NCs/NF electrode. On the basis of the above discussions, Ni(OH)_2_ NCs/NF electrode possesses significant advantages in electrochemical kinetics compared to Ni(OH)_2_ BNCs/NF.

### Electrochemical Performance of the ASC device

**Fig. 7:**
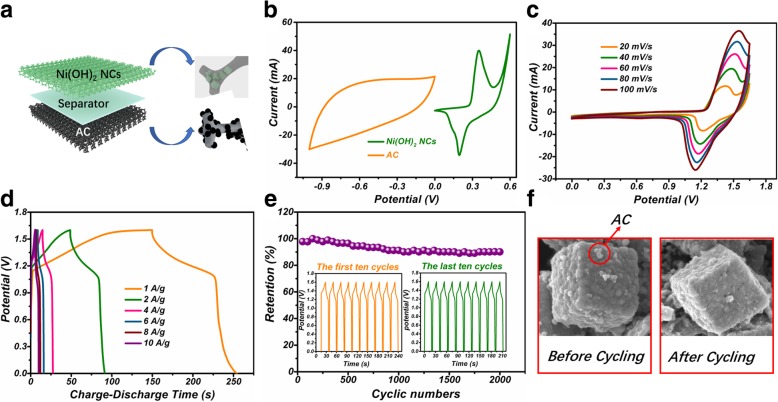
**a** Schematic of the Ni(OH)_2_ NCs/NF//AC device. **b** The CVs of AC and Ni(OH)_2_ NCs/NF electrodes in a three-electrode system. **c** The CVs of Ni(OH)_2_ NCs/NF//AC ASC between 0 and 1.6 V. **d** GCD curves of the ASC at different current densities between 0 and 1.6 V. **e** Cycling stability of the ASC during 2000 cycles at 4 A g^−1^. **f** The SEM images of the positive electrode before and after the cycling

The ASC device of Ni(OH)_2_ NCs/NF//AC was constructed according to Fig. 7a. Ni(OH)_2_ NCs/NF electrode and AC were separated by a cellulose paper. As illustrated in Fig. 7b, the CV curve of AC electrode presents nearly rectangular feature, revealing typical EDLC storage mechanism. Moreover, the AC electrode can be cycled within − 1 to 0 V and Ni(OH)_2_ NCs/NF electrode can be cycled within 0 to 0.6 V, revealing that the ASC device can afford an operation voltage of 1.6 V. The CV curves displayed in Fig. 7c show a well-defined shape even at high scan rates, implying excellent mass transport kinetics and eminent reversibility. GCD curves of the ASC device at different current densities were shown in Fig. 7d. The energy density and power density of the device were calculated according to Fig. 7d. An energy density of 23.3 Wh Kg^−1^ is achieved at a power density of 800 W Kg^−1^. An energy density of 9.6 Wh Kg^−1^ is still obtained even at a high power density of 8000 W Kg^−1^. The energy density is much larger than that of Ni(OH)_2_ BNCs/NF//AC ASC (Additional file 1: Figure S8, 3 Wh Kg^−1^ at 880 W Kg^−1^). Furthermore, the maximum energy density of the ASC is also larger than those of Ni(OH)_2_-based materials [31, 32]. The cycling stability was estimated by repeating GCD measurements at 4 A g^−1^ for 2000 cycles. The final specific capacitance still retains 90.1% of its largest value and this value is much larger than that of Ni(OH)_2_ BNCs/NF//AC ASC (Additional file 1: Figure S9, 60%). In addition, the last ten GCD curves are similar to the first ten cycles, exhibiting excellent stability of the ASC device. As shown in Fig. 7f, Ni(OH)_2_ NCs still retain the uniform cubic cage-like morphology after 2000 cycles, further demonstrating the excellent cycling stability. The loss of the specific capacitance may be attributed to the small amount of active material dropping from NF.

## Conclusions

Overall, Ni(OH)_2_ NCs were successfully constructed through a CEP method and used as an electrode for supercapacitors. Ni(OH)_2_ NCs present a large specific surface area of 54.7 m^2^/g and a concentrated pore size distribution between 2.7 and 6.1 nm. The thin shell shortens the transfer route and improves the electron transfer rate. As a positive electrode for supercapacitors, Ni(OH)_2_ NCs/NF displays a specific capacitance of 539.8 F g^−1^ at 1 A g^−1^, which is much larger than that of Ni(OH)_2_ BNCs/NF//AC (87.3 F g^−1^ at 1 A g^−1^). The specific capacitance still retains about 96.9% of its initial value after 2000 cycles. The ASC of Ni(OH)_2_ NCs/NF//AC possesses an energy density of 23.3 Wh Kg^−1^ at 800 W Kg^−1^, which is much larger than that of Ni(OH)_2_ BNCs (3 Wh Kg^−1^ at 880 W Kg^−1^). The results demonstrate that the designed Ni(OH)_2_ NCs have potential applications in the field of energy storage.

## Additional Files

Additional file 1: **Figure S1.** SEM image of the Ni(OH)_2_ BNCs sample. **Figure S2.** (a) XRD pattern and (b) SEM image of Cu_2_O templates. **Figure S3.** The relationship between peak current and square root of scan rates. **Figure S4.** The relationship between specific capacitance and charging-discharging current densities for Ni(OH)_2_ NCs/NF. **Figure S5.** (a) The GCD curves of Ni(OH)_2_ BNCs/NF at different current densities; (b) The relationship between specific capacitance and current densities for Ni(OH)_2_ BNCs/NF. **Figure S6.** The cycling stability of Ni(OH)_2_ BNCs/NF. **Figure S7.** The equivalent circuit of EIS. **Figure S8.** The GCD curves of Ni(OH)_2_ BNCs/NF//AC at different current densities. **Figure S9.** The cycling stability of Ni(OH)_2_ BNCs/NF//AC. (DOC 2752 kb)

## Data Availability

The datasets are available without restriction.

## Abbreviations

AC: Activated carbon
ASC: Asymmetric supercapacitor
BNCs: Broken nanocages
CEP: Coordinating etching and precipitating
CV: Cyclic voltammetry
EDLCs: Electric double-layer capacitors
EIS: Electrochemical impedance spectroscopy;
FESEM: Field emission scanning electron microscope
GCD: Galvanostatic charge-discharge
NCs: Nanocages
NF: Ni foam
PCs: Pseudocapacitors
PTFE: Polytetrafluoro ethylene
PVP: Polyvinyl pyrrolidone
TEM: Transmission electron microscope
XPS: X-ray photoelectron spectrometer
XRD: X-ray diffraction
