# Supplementary material for: Synthesis of Cubic Ni(OH)2 Nanocages Through Coordinating Etching and Precipitating Route for High-Performance Supercapacitors
Source: Nanoscale Res Lett. 2019 Aug 2;14:264. doi: 10.1186/s11671-019-3096-6 (PMC6890925; doi:10.1186/s11671-019-3096-6)
Supplement: Supplementary file 1 — Figure S1. SEM image of the Ni(OH)2 BNCs sample. Figure S2. (a) XRD pattern and (b) SEM image of Cu2O templates. Figure S3. The relationship between peak current and square root of scan rates. Figure S4. The relationship between specific capacitance and charging-discharging current densities for Ni(OH)2 NCs/NF. Figure S5. (a) The GCD curves of Ni(OH)2 BNCs/NF at different current densities; (b) The relationship between specific capacitance and current densities for Ni(OH)2 BNCs/NF. Figure S6. The cycling stability of Ni(OH)2 BNCs/NF. Figure S7. The equivalent circuit of EIS. Figure S8. The GCD curves of Ni(OH)2 BNCs/NF//AC at different current densities. Figure S9. The cycling stability of Ni(OH)2 BNCs/NF//AC. (DOC 2752 kb) [file 11671_2019_3096_MOESM1_ESM.doc]

**Synthesis of cubic Ni(OH)_2_ nanocages through coordinating etching and precipitating route for high performance supercapacitors Supporting Information**

Liangliang Tian^1,*^, Tong Yang^2^, Wanrong Pu^3^, Jinkun Zhang^4^

^1^ Research Institute for New Materials Technology, Chongqing University of Arts and Sciences, Chongqing, PR China

^2^ Faculty of Materials and Energy, Southwest University, Chongqing, PR China

^3^ School of Pharmacy, Tianjin Medical University, Tianjin, PR China

^4^ College of Pharmaceutical Science, Zhejiang University of Technology, Zhejiang, PR China

^*^Corresponding author at: Research Institute for New Materials Technology, Chongqing University of Arts and Sciences, Chongqing, PR China

E-mail addresses: tyty216216@163.com (LL Tian)

**This document file includes:**

Supplementary Figure S1 to S9


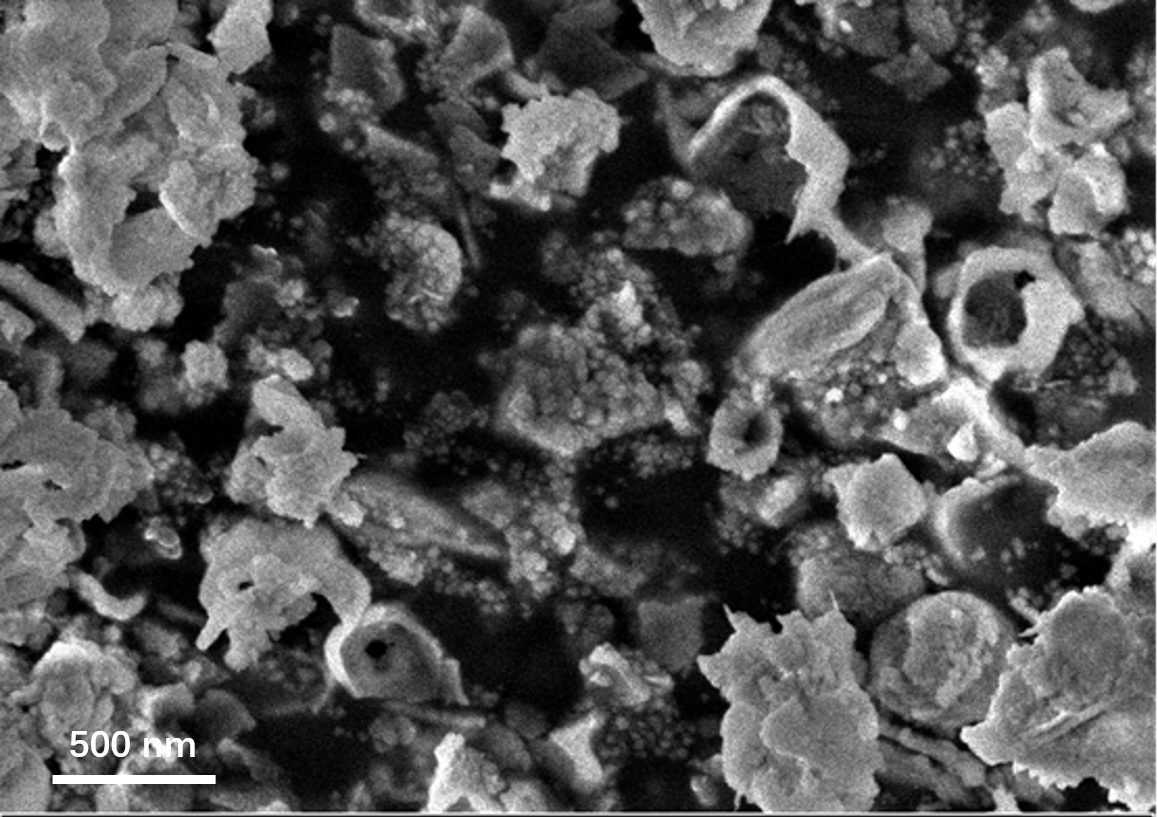


Fig. S1. SEM image of the Ni(OH)_2_ BNCs sample.


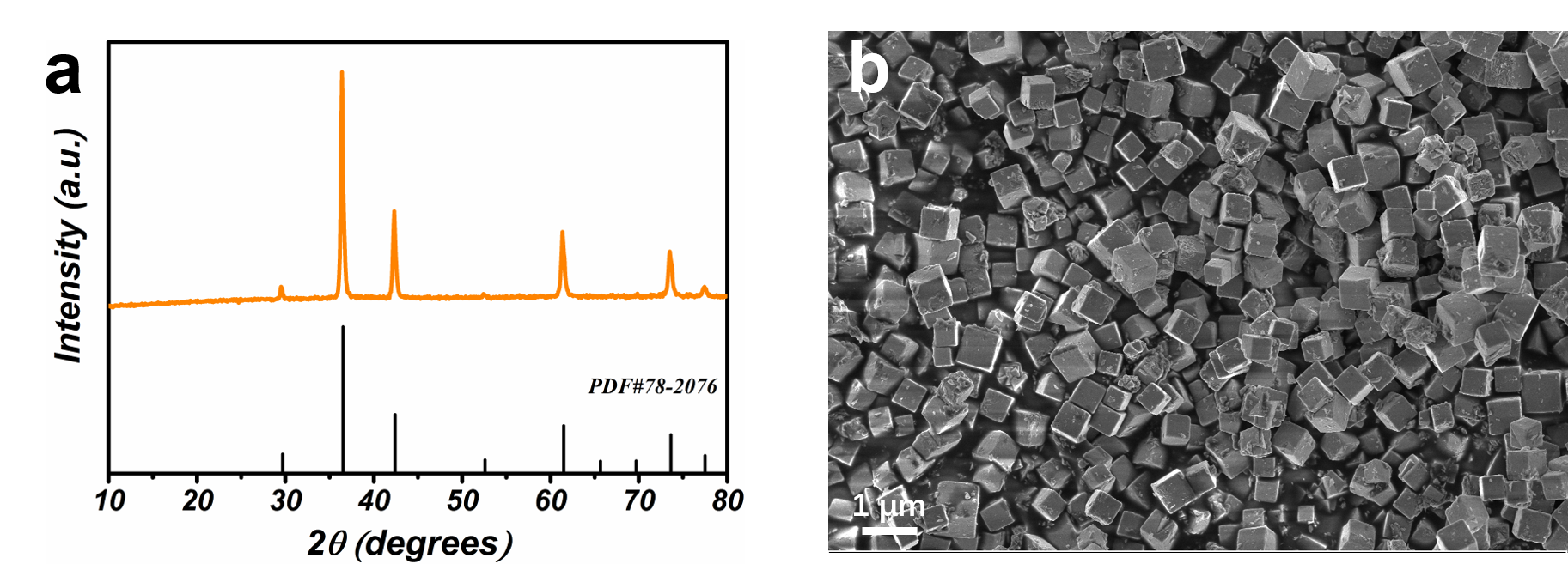


Fig. S2. (a) XRD pattern and (b) SEM image of Cu_2_O templates.


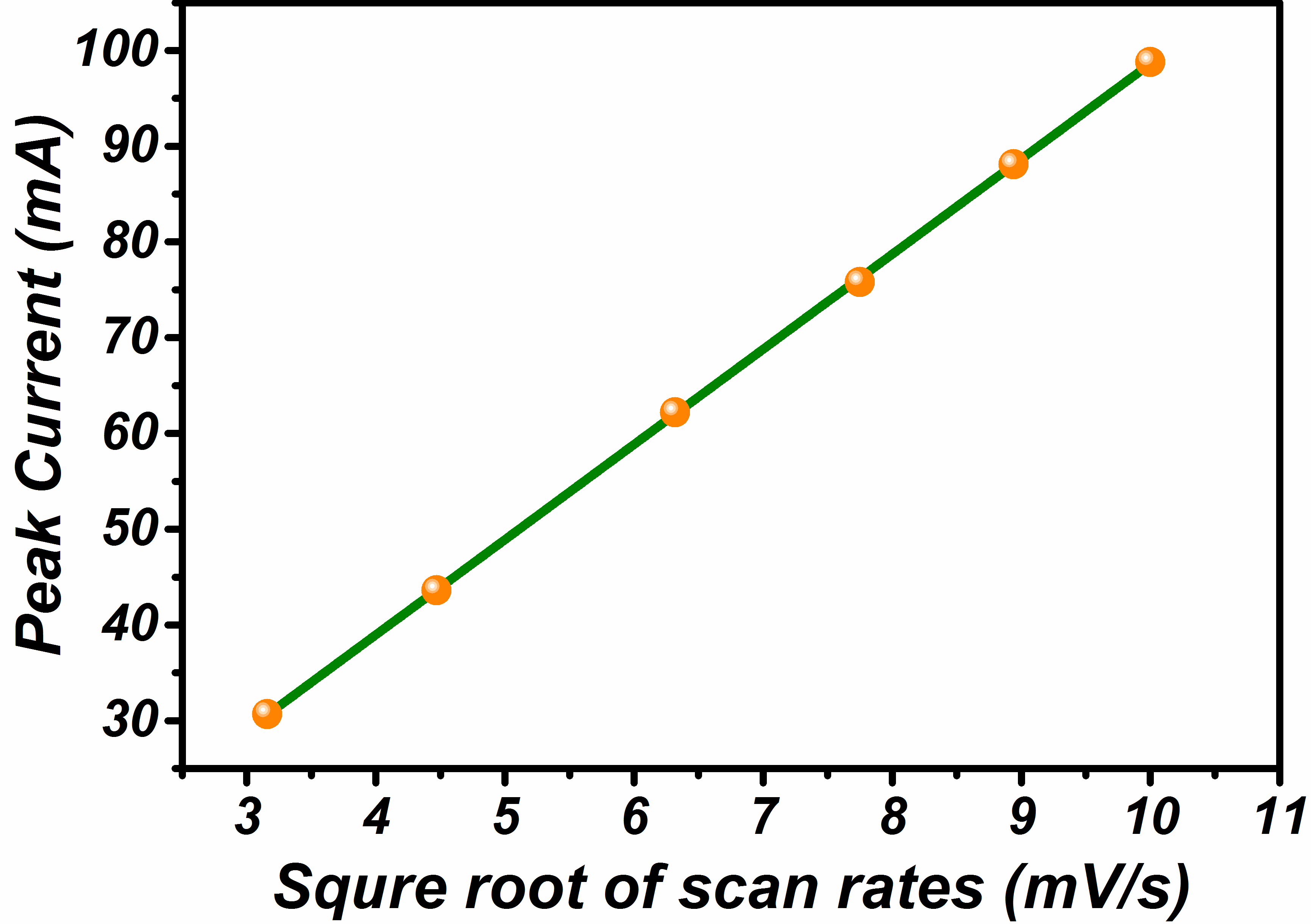
 Fig. S3. The relationship between peak current and square root of scan rates.


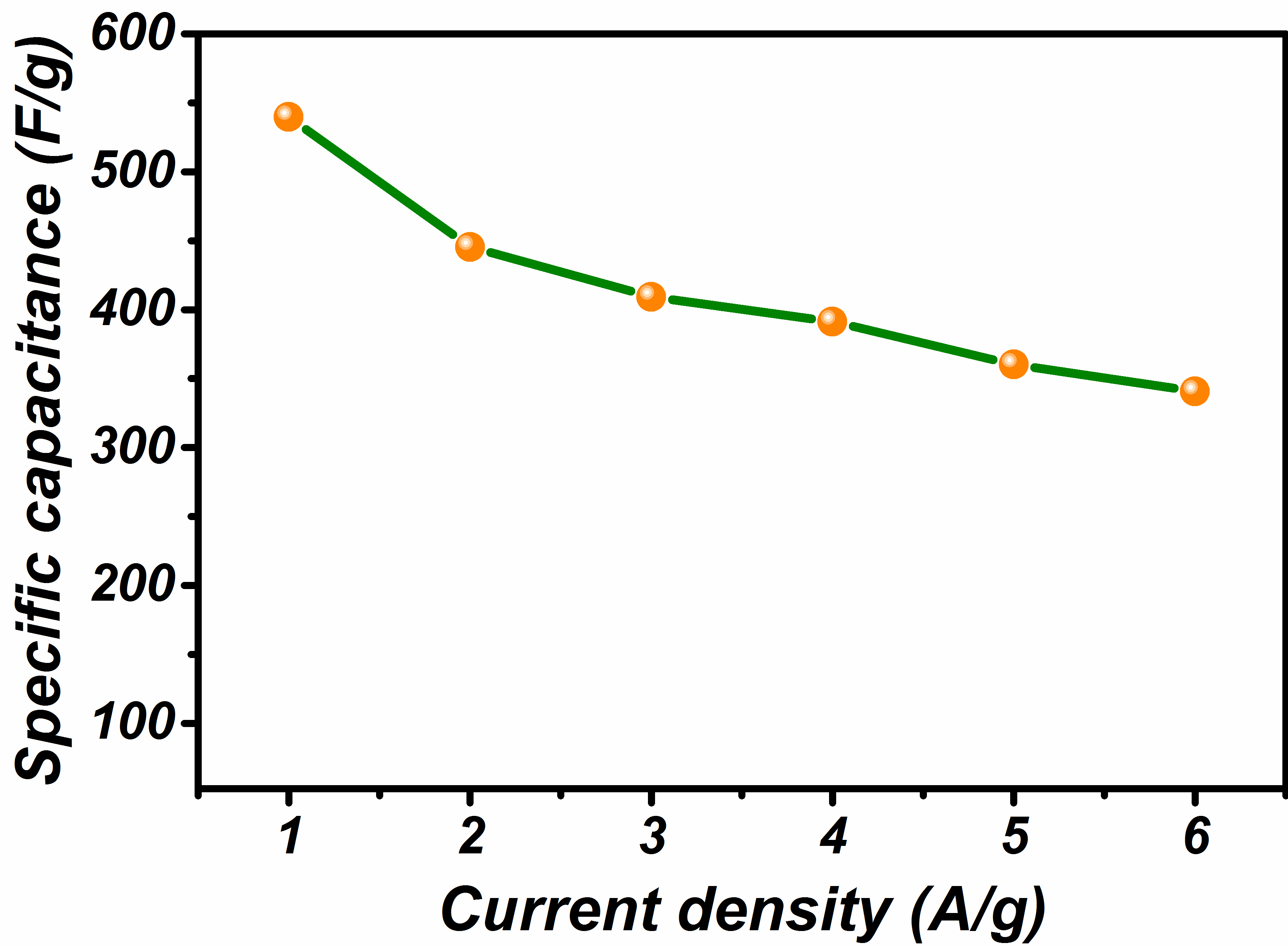
Fig. S4. The relationship between specific capacitance and charging-discharging current densities for Ni(OH)_2_ NCs/NF.


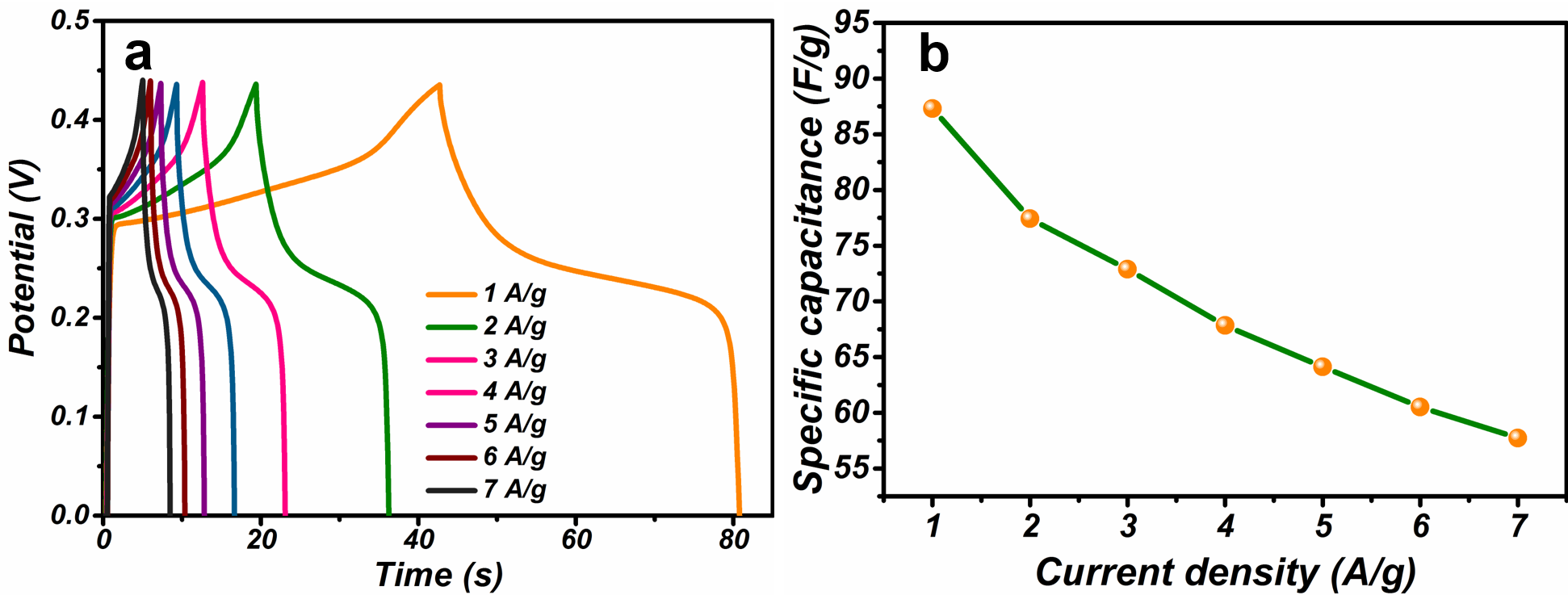
Fig. S5. (a) The GCD curves of Ni(OH)_2_ BNCs/NF at different current densities; (b) The relationship between specific capacitance and current densities for Ni(OH)_2_ BNCs/NF.


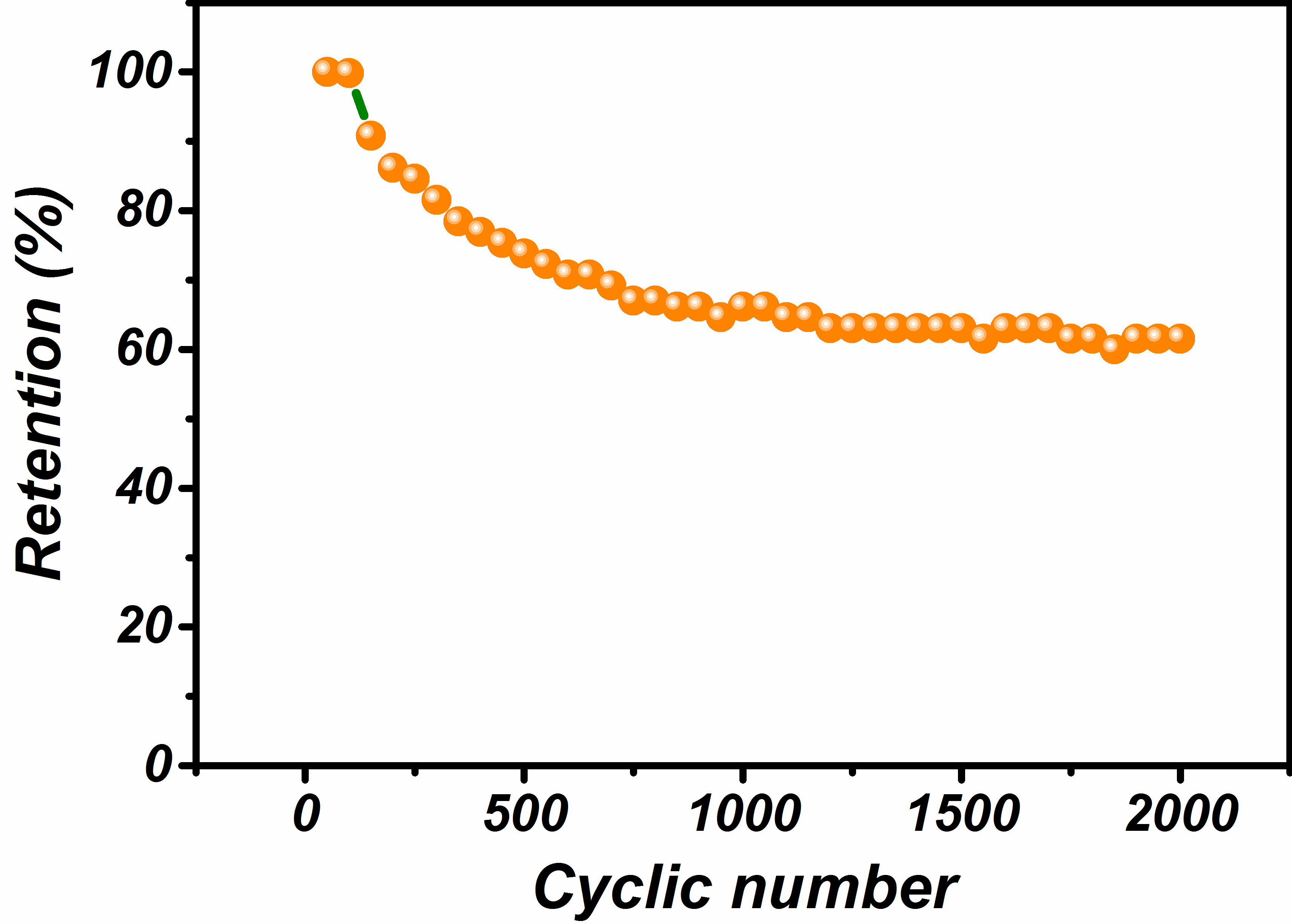
Fig. S6. The cycling stability of Ni(OH)_2_ BNCs/NF.


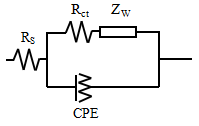


Fig. S7. The equivalent circuit of EIS.


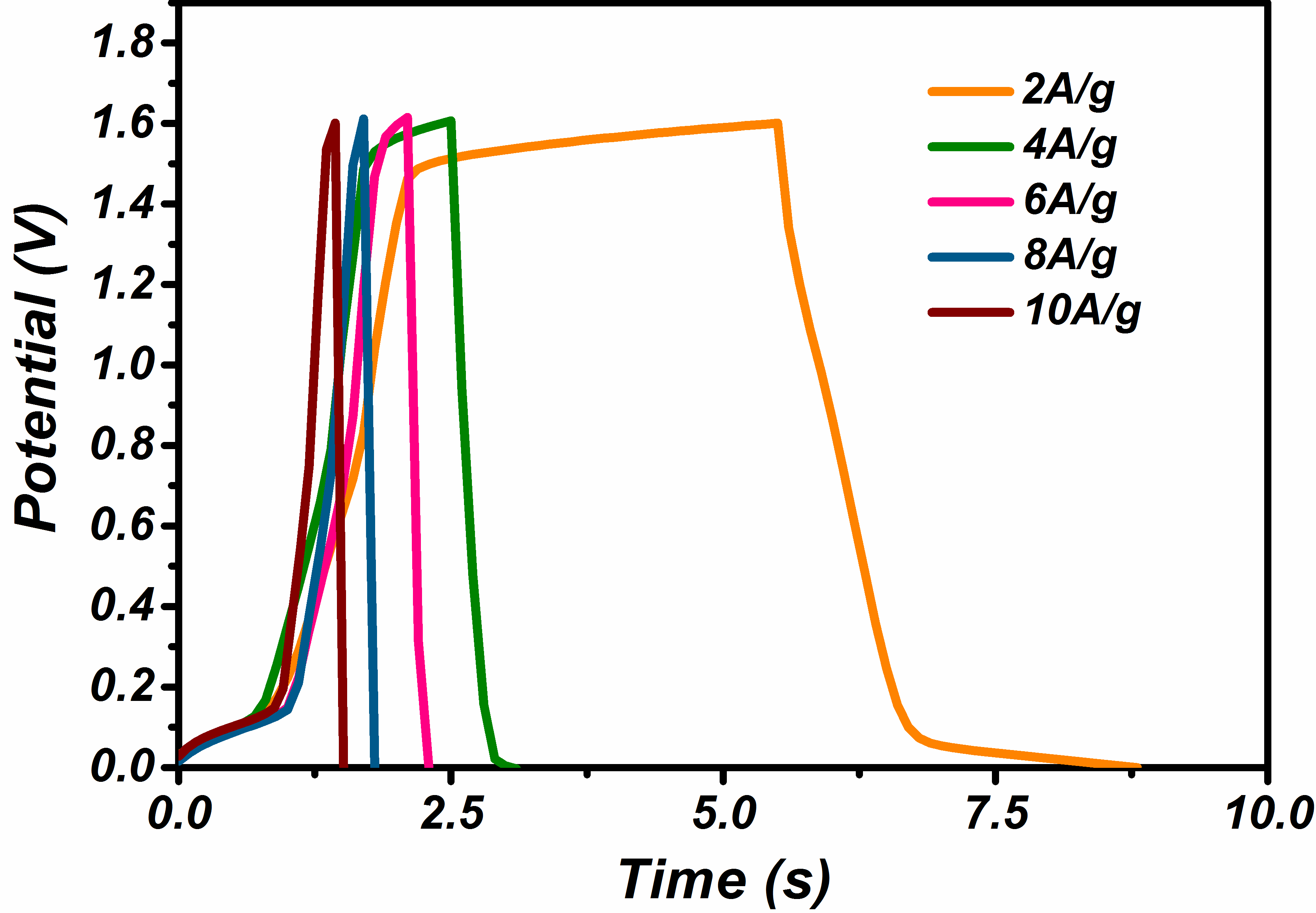
Fig. S8. The GCD curves of Ni(OH)_2_ BNCs/NF//AC at different current densities.


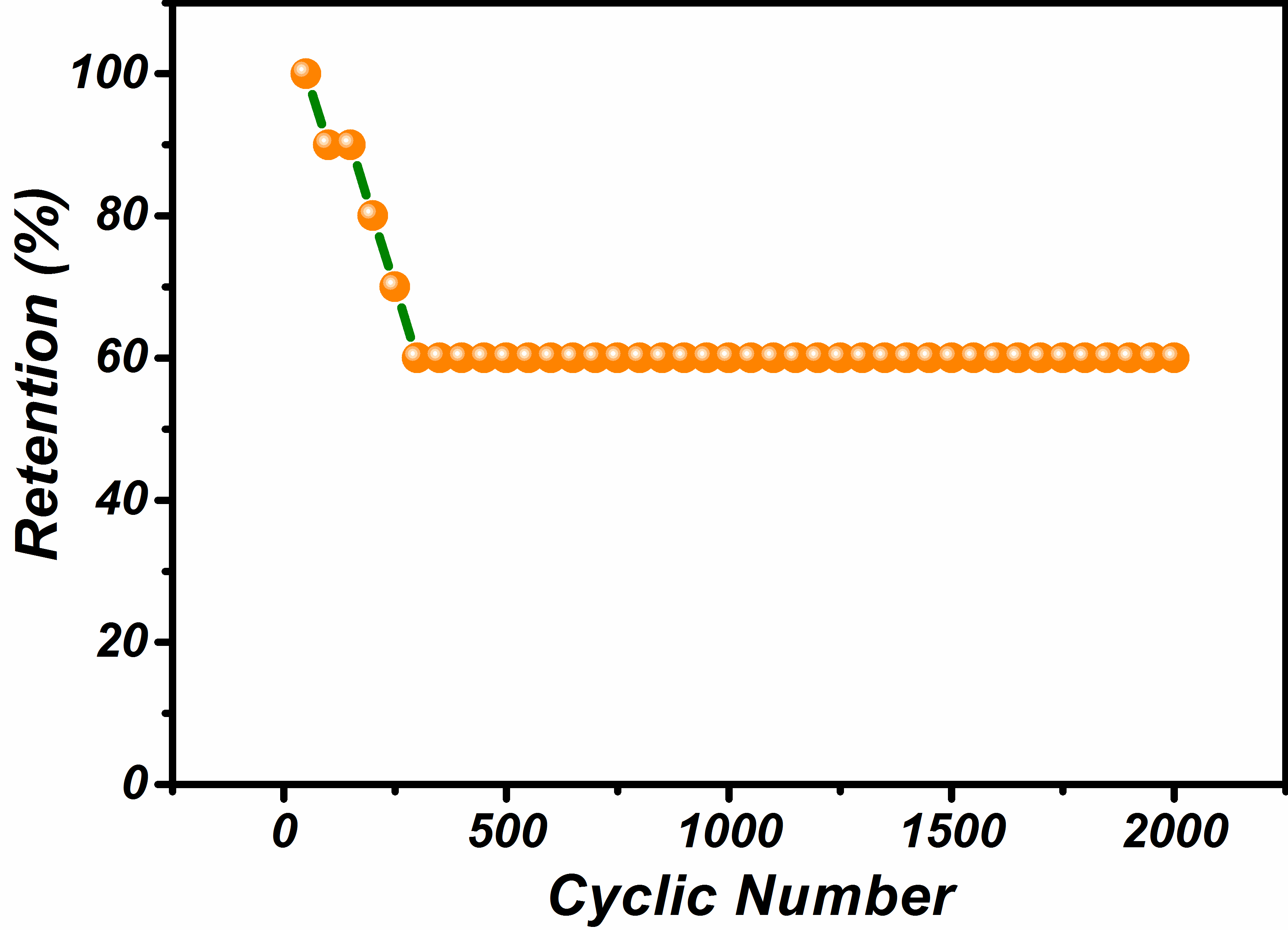


Fig. S9. The cycling stability of Ni(OH)_2_ BNCs/NF//AC.
